# Biomarkers for immune checkpoint inhibition in sarcomas – are we close to clinical implementation?

**DOI:** 10.1186/s40364-023-00513-5

**Published:** 2023-08-23

**Authors:** Chin Sern Yiong, Tzu Ping Lin, Vivian Yujing Lim, Tan Boon Toh, Valerie Shiwen Yang

**Affiliations:** 1 Translational Precision Oncology Laboratory, Institute of Molecular and Cell Biology (IMCB), Agency for Science, Technology and Research (A*STAR), Singapore, 138673 Singapore; 2 Present Address: Department of Pharmacy, National University of Singapore, Singapore, 117544 Singapore; 3 The N.1 Institute for Health, National University of Singapore, Singapore, Singapore; 4 The Institute for Digital Medicine (WisDM), National University of Singapore, Singapore, Singapore; 5 Division of Medical Oncology, National Cancer Centre Singapore, Singapore, 169610 Singapore; 6 Duke-NUS Medical School, Oncology Academic Clinical Program, Singapore, 169857 Singapore

**Keywords:** Immune checkpoint inhibitors, Predictive biomarkers, Sarcomas, Tertiary lymphoid structures

## Abstract

Sarcomas are a group of diverse and complex cancers of mesenchymal origin that remains poorly understood. Recent developments in cancer immunotherapy have demonstrated a potential for better outcomes with immune checkpoint inhibition in some sarcomas compared to conventional chemotherapy. Immune checkpoint inhibitors (ICIs) are key agents in cancer immunotherapy, demonstrating improved outcomes in many tumor types. However, most patients with sarcoma do not benefit from treatment, highlighting the need for identification and development of predictive biomarkers for response to ICIs. In this review, we first discuss United States (US) Food and Drug Administration (FDA)-approved and European Medicines Agency (EMA)-approved biomarkers, as well as the limitations of their use in sarcomas. We then review eight potential predictive biomarkers and rationalize their utility in sarcomas. These include gene expression signatures (GES), circulating neutrophil-to-lymphocyte ratio (NLR), indoleamine 2,3-dioxygenase (IDO), lymphocyte activation gene 3 (LAG-3), T cell immunoglobin and mucin domain-containing protein 3 (TIM-3), *TP53* mutation status, B cells, and tertiary lymphoid structures (TLS). Finally, we discuss the potential for TLS as both a predictive and prognostic biomarker for ICI response in sarcomas to be implemented in the clinic.

## Background

Sarcomas are a diverse and complex group of cancers of mesenchymal origin that often have very poor prognosis, with median survival of about 18 months with metastatic disease [1]. In soft-tissue sarcomas (STS), the 5-year survival rates for localized, regional, and metastatic disease are 81%, 56% and 16% respectively [2]. Comparatively, in osteosarcoma, the 5-year survival rates are 74%, 66% and 27% respectively [3]. Lastly, the 5-year survival rates in Ewing sarcoma are 81%, 67% and 38% respectively [4]. The systemic treatment of sarcomas has relied on conventional chemotherapy that has remained widely unchanged over several decades. Doxorubicin and ifosfamide represent the current standard of care in most subtypes of advanced and metastatic sarcomas [5]. However, response to treatment remains poor and more efficacious treatment options are needed. In a phase III trial comparing doxorubicin monotherapy against intensified doxorubicin with ifosfamide in advanced or metastatic STS, treatment with doxorubicin alone yielded an overall response rate of 14%, compared to 26% in patients treated with doxorubicin and ifosfamide. Importantly, there was no significant difference in overall survival (OS) between the two groups, with a median OS of 12.8 months (95.5% confidence interval (CI), 10.5–14.3) in the doxorubicin-only group, compared to 14.3 months (95.5% CI, 12.5–16.5) in the combination group [6]. Alternative agents such as gemcitabine and docetaxel are reserved for patients who have failed or are unable to tolerate doxorubicin and ifosfamide. Gemcitabine is commonly used alone or in combination with docetaxel, with complete or partial response, or stable disease after at least 25 weeks being achieved by 27% in the gemcitabine-only group and 32% in the combination group [7]. These response rates are in stark contrast to other tumors such as lymphomas, leukemias, germ cell tumors and others with response rates of > 70% with chemotherapy [8]. While targeted therapies are available, only less than 5% of STS are amenable to these treatments [9–11]. Limited treatment options compounded by poor treatment response necessitates the exploration of more treatment options with better outcomes and side effect profiles.

Research in treatment for sarcomas has faced many challenges. Sarcomas are rare cancers representing only 1% of adult malignancies [12], making it difficult to recruit sufficient clinical trial participants to generate rapid and robust evidence for treatment efficacy. Furthermore, heterogeneity in their histology and genetic drivers of oncogenic pathways in sarcomas gives rise to a wide variation in their biology, as well as degree of immune infiltration. As such, each subtype exhibits different clinical characteristics, often requiring patient-specific treatment approaches [13] since different patients may not respond to the same therapy.

Amidst these challenges, immune checkpoint inhibitor (ICI) therapy has emerged as an attractive treatment option [14]. ICIs target immune checkpoints that under physiologic conditions restrict the strength and duration of immune responses to avoid immune-mediated tissue damage, but which can be exploited by tumors to evade immune-mediated elimination. Efficacy of treatment with ICIs has been established in several cancers [15], including advanced renal cell carcinoma (RCC) [16], cervical cancer [17], classical Hodgkin lymphoma [18], gastric carcinoma [19], hepatocellular carcinoma (HCC) [20], melanoma [21–23], Merkel cell carcinoma [24, 25], non-small cell lung cancer (NSCLC) [26], primary mediastinal large B-cell lymphoma [27], small cell lung cancer [28], head and neck squamous cell cancer (HNSCC) [29], triple negative breast cancer [30], and urothelial cancer [31]. In an exciting step forward in the treatment of sarcoma, the United States (US) Food and Drug Administration (FDA) recently approved the first ICI for use in the treatment of STS, with atezolizumab being approved for use in the treatment of unresectable or metastatic alveolar soft-part sarcomas (ASPS) [32]. Atezolizumab as the first agent of its class being indicated for ASPS could set the stage for more ICIs to be indicated for the treatment of more STS subtypes and offers exciting possibilities for further evaluation.

In fact, although STS have been traditionally thought to be immune “cold” [33], as a whole, the response of STS to immune checkpoint inhibition does not differ too much from that of all cancers considered together. In 2019, Haslam and Prasad estimated that the percentage of US patients with cancer that respond to ICIs was 12.46% (95% CI, 12.37–12.54%) [34], which is comparable to the results of the SARC028 trial (NCT02301039), where 18% of patients with STS had an objective response to pembrolizumab [35]. Additionally, ICI therapy has shown improved outcomes in the clinical management of selected populations in sarcomas [36–38]. Within STS subtypes, liposarcomas (LPS), undifferentiated pleomorphic sarcomas (UPS) and ASPS have demonstrated better responses than other subtypes, while leiomyosarcomas (LMS) and synovial sarcomas (SS) have been reported to be resistant to ICI monotherapy [39]. Table 1 outlines a comprehensive list of studies using ICIs, both as monotherapy and in combination, and the respective clinical outcomes in sarcomas. Aside from clinical efficacy, another concern that clinicians have to consider is the potential for immune-related adverse events (irAEs) that range from mild adverse conditions like diarrhea and rashes to life-threatening conditions like cardiomyopathy and toxic epidermal necrolysis [40]. Thus, there is an urgent need to identify biomarkers that can guide clinical use of ICIs in potential responders while sparing non-responders from potentially life-threatening irAEs.

**Table 1:** Overview of studies using immune checkpoint inhibitors (ICIs) alone or in combination with other drugs in sarcomas

**ICI**	**Combination**	**NCT**	**Phase (Status)**	**Type of Tumor**	**Clinical Efficacy**	** ≥ G3 TRAE**
Atezolizumab	Cabozantinib	NCT05019703	Phase II (recruiting)	OGS	NA	NA
	± CMB305	NCT02609984	Phase II (terminated due to failure to meet efficacy objective)	NY-ESO-1 + sarcoma	Atezolizumab only: 0 CR, 0 PR, 17 SD, 25 PD (*n* = 44) Atezolizumab + CMB305: 1.8% ORR (95%CI: 0.8–4.2%), 0 CR, 1 PR, 23 SD, 19 SD (*n* = 45) mPFS: 1.6 months in atezolizumab only arm (*n* = 43), 2.6 months in atezolizumab + CMB305 arm (*n* = 45) (HR: 0.9, 95% CI: 0.6–1.3) mOS: 18 months in both arms (atezolizumab only arm: 95% CI, 15.3 to 26.5 and atezolizumab + CMB305 arm: 95% CI, 10.1 to 22.1; HR, 1.2; *p* = 0.47)	13 ≥ G3 TRAE reported in atezolizumab only arm 18 ≥ G3 TRAE reported in atezolizumab + CB305 arm
	Bevacizumab	NCT03141684	Phase II (recruiting)	ASPS	1 CR, 14 PR, 1 unconfirmed PR, 25 SD (*n* = 43)	10 ≥ G3 TRAE
	Bevacizumab + rucaparib	NCT03694262	Phase II (active, not recruiting)	Endometrial cancer, uterine carcinosarcoma	1 CR, 9 PR, 13 SD (*n* = 26)	≥ G3 TRAE reported in 50% patients
	Cobimetinib	NCT04216953	Phase I/II (recruiting)	STS	NA	NA
	Irinotecan + temozolomide + vincristine	NCT04796012	Phase I/II (recruiting)	Rhabdomyosarcoma, solid tumor	NA	NA
	NA	NCT04273061	Phase II (recruiting)	Cancers (breast, gastrointestinal, genitourinary, gynecologic, head and neck, lung, skin, unknown primary tumor), sarcoma	NA	NA
		NCT04458922	Phase II (active, not recruiting)	Chondrosarcoma, clear cell sarcoma of soft tissue	3 SD (*n* = 9 in grade 2/3 chondrosarcoma cohort) No RECIST objective responses observed (*n* = 9 in dedifferentiated chondrosarcoma cohort)	Grade 3 TRAEs occurred in 2 patients in dedifferentiated chondrosarcoma cohort (22%), included infusion reaction, myonecrosis, and anemia
	RT + surgical resection	NCT03474094	Phase II (recruiting)	STS	NA	NA
	SABR	NCT02992912	Phase II (unknown)	Metastatic tumors (colorectal cancer, NSCLC, RCC, sarcoma)	NA	NA
	Selinexor	NCT05333458	Phase II (recruiting)	ASPS, STS	NA	NA
	Tiragolumab	NCT05286801	Phase I/II (recruiting)	Epithelioid sarcoma, SMARCB1 or SMARCA4 deficient tumors	NA	NA
	Tivozanib	NCT05000294	Phase I/II (recruiting)	Bile duct cancer, breast cancer, gall bladder cancer, neuroendocrine cancer, ovarian cancer, pancreatic adenocarcinoma, prostate cancer, STS, vulvar cancer	NA	NA
± Atezolizumab	SBRT	NCT03548428	Phase II (recruiting)	Sarcoma	NA	NA
Avelumab	NA	NCT03006848	Phase II (active, not recruiting)	OGS	No objective responses occurred (17 PD) (*n* = 18) mPFS: 8 weeks (95% CI: 6.7–9.1 months)	6 ≥ G3 TRAE
	Trabectedin	NCT03074318	Phase I/II (terminated due to investigator leaving institute)	LMS, LPS	2 DLT reported (*n* = 6) 2 PR (1 confirmed), 11 SD (*n* = 23) mPFS: 23.4 months	Most common G3 TRAE attributed to study drug were neutropenia and ALT increase No G4/5 TRAE at the Phase 2 dose
Camrelizumab	Apatinib	NCT04239443	Phase II (unknown)	NSCLC, STS, uterine cancer	NA	NA
	Cisplatin + doxorubicin + ifosfamide + methotrexate	NCT04294511	Phase II (recruiting)	OGS	31 showed good response (*n* = 65)	Most common grade 3–4 adverse events were decreased platelet count (44.0%), decreased white blood cell (37.3%), decreased neutrophil count (29.3%), oral mucositis (14.7%), increased alanine aminotransferase (12.0%), and increased aspartate aminotransferase (10.7%)
	Ifosfamide + liposome doxorubicin	NCT04606108	Phase II (recruiting)	STS	NA	NA
± Camrelizumab	Famitinib ± ifosfamide	NCT04044378	Phase I/II (withdrawn due to toxicity)	OGS	NA	NA
Durvalumab + ipilimumab + pembrolizumab	NA	NCT05187338	Phase I/II (recruiting)	Sarcoma, solid tumors	NA	NA
Envafolimab ± ipilimumab	NA	NCT04480502	Phase II (recruiting)	MFS, UPS	NA	NA
Envafolimab + YH001 (anti-CTLA4 antibody)	± Doxorubicin	NCT05448820	Phase I/II (recruiting)	Sarcoma	NA	NA
FAZ053 (anti-PD-L1 antibody) ± spartalizumab	NA	NCT02936102	Phase I (active, not recruiting)	ASPS, chordoma, solid tumors, TNBC	NA	NA
Ipilimumab	CD4^+^ T cells + cyclophosphamide	NCT02210104	Phase I (withdrawn due to issues with tetramer staining)	Melanoma, sarcoma	NA	NA
	Dasitinib	NCT01643278	Phase I (completed)	GIST, STS	DLT included grade 3 gastric hemorrhage and anemia 0 CR, 0 PR (*n* = 28) mPFS: 2.8 months (95% CI: 2.7–3.0 months) (*n* = 18) mOS: 13.5 months (95% CI: 11.4 months – NR)	19 ≥ G3 TRAE
	NA	NCT00140855	Phase II (terminated due to poor accrual)	SS	0 CR, 0 PR, 0 SD, 6 PD (*n* = 6)	3 ≥ G3 TRAE
		NCT01445379	Phase I (completed)	Lymphoma, neuroblastoma, sarcoma, Wilms’ tumor	DLT observed at 10 mg/kg (*n* = 2) 6 SD for four to ten cycles (clear cell sarcoma, melanoma, OGS, SS)	11 ≥ G3 TRAE
Ipilimumab + nivolumab	Cabozantinib	NCT04149275	Phase II (withdrawn due to stoppage of funding by sponsor)	Gynecologic carcinosarcoma	NA	NA
		NCT04551430	Phase II (active, not recruiting)	STS	NA	NA
	± Cabozantinib	NCT05836571	Phase II (not yet recruiting)	Extraskeletal myxoid chondrosarcoma, LMS, LPS, UPS	NA	NA
	Cryoablation	NCT04118166	Phase II (active, not recruiting)	STS	0 CR, 3 PR, 7 SD, 19 PD (*n* = 29)	41 ≥ G3 TRAE
		NCT05302921	Phase II (recruiting)	ES, hepatoblastoma, hepatocellular carcinoma, melanoma, neuroblastoma, OGS, rhabdomyosarcoma, Wilms’ tumor	NA	NA
	Lurbinectedin	NCT05876715	Phase II (recruiting)	STS	NA	NA
	NA	NCT02982486	Phase II (unknown)	BS, STS	NA	NA
		NCT03219671	Phase II (unknown)	Classic Kaposi sarcoma	87% ORR (*n* = 15)	2 ≥ G3 TRAE
		NCT04416568	Phase II (recruiting)	Epithelioid sarcoma, INI1-negative cancers	NA	NA
		NCT04465643	Phase I (recruiting)	MPNST	NA	NA
	Pazopanib alone	NCT04741438	Phase III (recruiting)	Sarcoma	NA	NA
	Tazemetostat	NCT05407441	Phase I/II (recruiting)	INI1-negative/SMARCA4-deficient cancers	NA	NA
	Trabectedin	NCT03138161	Phase I/II (recruiting)	STS	8 CR, 11 PR, 58 SD and 11 PD with 21.6% BORR and 87.5% DCR (*n* = 88) mPFS: 7 months (1–44 months) mOS: 14 months (1–46 months)	76 ≥ G3 TRAE
± Ipilimumab	XmAb23104	NCT03752398	Phase I (recruiting)	Solid tumors, UPS	No DLT reported (*n* = 62) 3 PR in HNSCC, RCC, sarcoma	≥ G3 TRAE reported in 6 patients 2 ≥ G3 irAEs
± Ipilimumab or pembrolizumab	INT230-6	NCT03058289	Phase I/II (completed)	Cancer, sarcoma	No DLT reported	Incidence of ≥ G3 TRAE was 11% and 14% in INT230-6 only and INT230-6 + pembrolizumab arm 1 G4 neutrophil count decrease reported in INT230-6 + pembrolizumab arm
± Ipilimumab with nivolumab	Aldesleukin + autologous TIL LN-145 + autologous TIL LN-145-S1	NCT03449108	Phase II (recruiting)	Anaplastic thyroid cancer, BS, STS, relapsed/refractory ovarian cancer, TNBC, undifferentiated high grade pleomorphic sarcoma of bone	NA	NA
LAG525 + spartalizumab	NA	NCT03365791	Phase II (completed)	Solid and hematologic malignancies, STS	7.3% ORR (*n* = 75) mPFS: 2.8 months (95% CI: 2.6–3.1 months)	Serious adverse events in 35 patients reported (*n* = 76)
Nivolumab	Anlotinib hydrochloride	NCT04165330	Phase I/II (active, not recruiting)	NSCLC, SCLC, STS	NA	NA
	± Azacitidine	NCT03628209	Phase I/II (recruiting)	OGS, sarcoma	NA	NA
	Bempegaldesleukin	NCT03282344	Phase II (active, not recruiting)	Sarcoma	9 PR (*n* = 77) mPFS: 1.8–7.3 months mOS: 5.9–21.7 months (NR in ASPS and angiosarcoma)	32 ≥ G3 TRAE 1 possible treatment related death
		NCT04730349	Phase I/II (terminated due to changes in business objectives)	ES, recurrent/treatment-resistant cancers	NA	NA
	BMS-986205	NCT04106414	Phase II (closed to accrual due to lack of observed clinical efficacy)	Endometrial adeno-, carcino-sarcoma	No response in nivolumab only arm (*n* = 12) 1 PR in nivolumab + BMS-986205 arm (*n* = 12) mPFS: 7.3 weeks (80% CI: 6.4–15.1 weeks) (nivolumab only), 12.3 weeks (80% CI: 4.1–22.1 weeks) (nivolumab + BMS-986205) mOS: 27.5 weeks (80% CI: 17-NA) (nivolumab only), NR (nivolumab + BMS-986205)	3 ≥ G3 TRAE in nivolumab only arm 2 ≥ G3 TRAE in nivolumab + BMS-986205 arm
	BO-112 + RT + surgical resection	NCT04420975	Phase I (active, not recruiting)	STS	NA	NA
	Cabozantinib	NCT04514484	Phase I (recruiting)	Advanced cancer, HIV, Kaposi sarcoma	NA	NA
	± Cabozantinib S-malate or paclitaxel or paclitaxel only	NCT04339738	Phase II (active, not recruiting)	Angiosarcoma	Taxane only: 13 PR (*n* = 21), 13 ORR (*n* = 18) mPFS: 9.6 months (5.3 months – NR) mOS: 20.5 months (14.4 months – NR)	G3 hypertension reported in 10% patients only
	Cisplatin + dacarbazine + doxorubicin + epirubicin + ifosfamide + methotrexate + sunitinib	NCT03277924	Phase I/II (recruiting)	BS, STS	1 CR, 1 PR, 22 SD, 16 PD (*n* = 40) mPFS: 3.7 months (95% CI: 3.4–4 months) mOS: 14.2 months (95% CI: 7.1–21.3 months)	21 ≥ G3 TRAE
	Docetaxel + doxorubicin + gemcitabine	NCT04535713	Phase II (recruiting)	Sarcoma	8 PR, 44SD, 7 PD (*n* = 59 in intention-to-treat cohort) mPFS: 5.1 months (2.837–7.363 months) mOS: 15.3 months (95%CI: 5.48–25.12 months)	60 ≥ G3 TRAE
	NA	NCT03241745	Phase II (active, not recruiting)	Carcinosarcoma, clear cell carcinoma, endometrial carcinoma, high grade endometrial stromal sarcoma, LMS, undifferentiated sarcoma, uterine cancer	NA	NA
		NCT03316274	Phase I (completed)	HIV/AIDS, Kaposi sarcoma	NA	NA
		NCT03465592	Phase I/II (recruiting)	Sarcoma	NA	NA
		NCT05224999	Phase II (recruiting)	Carcinosarcoma	NA	NA
	Nab-rapamycin	NCT03190174	Phase I/II (completed)	Sarcoma and certain cancers	Two DLTs reported at 150 mg/m^2^ (grade 3 aspartate aminotransferase elevation and grade 4 thrombocytopenia) and 125 mg/m^2^ (grade 3 suicidal ideation and grade 3 hypophosphatemia) each (*n* = 26)	12 ≥ G3 TRAE
	± Pazopanib	NCT03149120	Phase II (withdrawn)	STS	NA	NA
	Pomalidomide	NCT04902443	Phase I (recruiting)	Kaposi sarcoma, viral Associated Malignancies	NA	NA
	Regorafenib	NCT04803877	Phase II (active, not recruiting)	OGS	NA	NA
	Rucaparib	NCT04624178	Phase II (active, not recruiting)	LMS	NA	NA
	Trabectedin	NCT03590210	Phase II (completed)	STS	mPFS: 5.5 months in LMS/LPS cohort (*n* = 43), 2.3 months in others (*n* = 49) mOS: 18.7 months in LMS/LPS cohort (*n* = 43), 5.6 months in others (*n* = 49)	NA
	Trabectedin + T-VEC	NCT03886311	Phase II (recruiting)	Sarcoma	3 PR, 30 SD, 6 PD, 7.7% BORR (*n* = 39) mPFS: 7.8 months (95% CI: 4.1–13.1 months) mOS: 19.3 months (95% CI: 12.8 months-NR)	3 ≥ G3 TRAE related to nivolumab 38 ≥ G3 TRAE related to trabectedin 1 ≥ G3 TRAE related to T-VEC
± Nivolumab	Bempegaldesleukin ± NKTR-262	NCT03435640	Phase I/II (terminated due to poor overall results)	CRC, HNSCC, melanoma, Merkel cell carcinoma, RCC, sarcoma, TNBC	1 DLT reported at 3.84 mg NKTR-262 2 PR (*n* = 17)	Most frequent treatment-related adverse events were flu-like symptoms, fatigue, nausea, and pruritus
	TPST-1120	NCT03829436	Phase I (active, not recruiting)	Advanced cancer, sarcoma	G3 hypertension reported in TPST-1120 monotherapy 3 G3 TRAE reported in combination therapy arm 10 SD (*n* = 19 in monotherapy arm)	3 ≥ G3 TRAE in combination therapy arm
Nivolumab ± Ipilimumab	NA	NCT02304458	Phase I/II (completed)	Lymphoma, recurrent/refractory solid tumors or sarcomas	No DLT reported (*n* = 12) Hodgkin lymphoma (*n* = 10): 1 CR, 2 PR, 5 SD Neuroblastoma (*n* = 10): 5 SD Sarcoma (*n* = 33): 11 SD	54 ≥ G3 TRAE
		NCT02428192	Phase II (active, not recruiting)	LMS	mPFS: 1.8 months (95% CI: 0.8 months – unknown) (*n* = 12) mOS: NR	14 ≥ G3 TRAE
		NCT02500797	Phase II (active, not recruiting)	Sarcoma	Nivolumab only: 3 PR, 5% ORR (92% CI:1–15%) (*n* = 38) Nivolumab + Ipilimumab arm: 15% adjusted ORR (92% CI: 6–30%) (*n* = 41) mPFS: 1.7 months (95% CI: 1.4–4.3 months) (*n* = 42 in nivolumab only arm), 4.1 months (95% CI: 2.6–4.7 months) (*n* = 41 in nivolumab + ipilimumab arm) mOS: 10.7 months (95% CI: 5.5–15.4 months) (*n* = 42 in nivolumab only arm), 14.3 months (95% CI: 9.6 months – not estimable) (*n* = 41 in nivolumab + ipilimumab arm)	44 ≥ G3 TRAE in nivolumab only arm 66 ≥ G3 TRAE in nivolumab + ipilimumab arm
	RT	NCT03463408	Phase I (active, not recruiting)	Sarcoma	NA	NA
	± RT	NCT03307616	Phase II (active, not recruiting)	DDLPS, UPS	mPFS: 18 months (IQR:8 months – NR in DDLPS), NR (IQR:19 – NR in UPS) mOS: NR	NA
Nivolumab ± relatlimab	NA	NCT04095208	Phase II (recruiting)	STS	NA	NA
ONC-392 (anti-CTLA4 IgG1 monoclonal antibody) ± pembrolizumab	NA	NCT04140526	Phase I/II (recruiting)	Sarcoma, solid tumors	NA	NA
± PD-1 inhibitor (not specified)	Anlotinib hydrochloride	NCT05193188	Phase II (recruiting)	Chondrosarcoma	NA	NA
	CAB-AXL-ADC	NCT03425279	Phase I/II (recruiting)	BS, ES, LMS, LPS, melanoma, NSCLC, OGS, refractory sarcoma, solid tumor, SS, STS	NA	NA
Pembrolizumab	Antiretroviral therapy	NCT02595866	Phase I (active, not recruiting)	HIV/AIDS related cancer, Kaposi sarcoma	NA	≥ G3 TRAE reported in 20% of patients
	APG-115	NCT03611868	Phase I/II (recruiting)	Melanomas, MPNST, solid tumors	Cutaneous/uveal melanoma:2 CR, 2 PR (*n* = 17) Melanoma: 2 CR, 3 PR (*n* = 38) MPNST: 4 SD (*n* = 10) LPS: 1 PR (*n* = 17)	≥ G3 TRAE reported in ≥ 5% patients
	Axitinib	NCT02636725	Phase II (completed)	STS	0 CR, 8 PR, 9 SD (*n* = 32) mPFS: 4.7 months in intention-to-treat analysis (95% CI: 3.0–9.4 months) (*n* = 33), 6.9 months in per-protocol analysis (95% CI: 3.0–9.4 months) (*n* = 30) mOS: 18.7 months (95% CI: 12.0 months – NR) (*n* = 33)	26 ≥ G3 TRAE
	Cabozantinib	NCT05182164	Phase II (recruiting)	ES, OGS, STS	NA	NA
	Cyclophosphamide	NCT02406781	Phase II (unknown)	Sarcoma	9 PR, 10 SD (*n* = 30) mPFS: 4.1 months (95%CI: 1.4–12.5 months) mOS: 18.3 months (95%CI: 8.5 months – NR)	9 ≥ G3 TRAE (*n* = 35)
	Cyclophosphamide + fludarabine	NCT03697824	Phase II (withdrawn due to internal decision, study will be replaced with a larger monotherapy trial)	NY-ESO-1 and/or LAGE-1a + SS	NA	NA
	Dactinomycin + melphalan	NCT04332874	Phase II (recruiting)	ASPS, myxofibrosarcoma, UPS	NA	NA
	Docetaxel + gemcitabine or + gemcitabine or gemcitabine + vinorelbine or irinotecan or liposomal doxorubicin	NCT02331251	Phase I/II (terminated as investigator is no longer at site)	Advanced cancer, sarcoma	2 DLT reported	≥ G3 TRAE reported in 12 patients (*n* = 17)
	Doxorubicin	NCT03056001	Phase II (completed)	STS	1 CR, 8 PR, 12 SD, 33% ORR (*n* = 27) mPFS: 6.9 months mOS: 15 months	26 ≥ G3 TRAE
	Doxorubicin hydrochloride	NCT02888665	Phase I/II (completed)	Sarcoma	No DLT reported Overall: 7 PR, 2 unconfirmed PR, 11 SD, 19% ORR (*n* = 37) Phase II: 4 PR (*n* = 31) mPFS: 8.1 months (95%CI: 7.6–10.8 months) mOS: 27.6 months (95%CI: 18.7%—NR)	24 ≥ G3 TRAE Notable pembrolizumab-related toxic effects included grade 3 adrenal insufficiency (*n* = 1) and hypothyroidism (*n* = 7)
	Epacadostat	NCT03414229	Phase II (active, not recruiting)	Sarcoma	1 PR, 47% DCR (CR + PR + SD) (*n* = 30) mPFS: 7.6 weeks (95% CI: 6.9–26.7 weeks) mOS: 16.9 weeks (95% CI: 9.4 weeks – not estimable)	7 ≥ G3 TRAE
	Eribulin	NCT03899805	Phase II (active, not recruiting)	LPS, LMS, UPS	1 PR, 5SD, 5.3% ORR (*n* = 19 in LMS cohort) mPFS: 11.1 weeks in LMS cohort	68% ≥ G3 TRAE in LMS cohort
	Gemcitabine	NCT03123276	Phase I/II (unknown)	LMS, UPS	DLT observed at gemcitabine 1000 mg/m^2^, but not confirmed in the expansion cohort LMS: 8 SD, 3 PD (*n* = 11) UPS: 2 PR (*n* = 2) mPFS: 5.1 months (95% CI: 2–9 months)	NA
	IFN-γ-1β	NCT03063632	Phase II (active, not recruiting)	Mycosis Fungoides and Sezary syndrome, myxoid LPS, round cell LPS, SS	NA	NA
	Lenvatinib	NCT04784247	Phase II (recruiting)	Sarcoma	NA	NA
		NCT05147558	Phase II (recruiting)	Uterine carcinosarcoma	NA	NA
		NCT05846724	Phase II (not yet recruiting)	Relapsed/refractory Kaposi sarcoma	NA	NA
	Modified vaccinia virus Ankara vaccine expressing p53	NCT02432963	Phase I (not recruiting)	Solid tumors, STS	1 DLT reported 3 SD (*n* = 11)	1 fatal G5 myocarditis reported 10 ≥ G3 TRAE
	NA	NCT02301039	Phase II (completed)	BS, STS	5.0% PR (95% CI: 71.0–16.9%) (*n* = 40 in BS), 17.5% PR (95% CI: 7.3–32.8%) (*n* = 40 in STS), 13.0% PR (95% CI: 5.5–25.3%) (*n* = 53 in expansion cohort) mPFS: 8 weeks (95% CI: 7–9 weeks) (*n* = 39 in BS), 18 weeks (95% CI: 8–22 weeks) (*n* = 37 in STS), 8 weeks (95% CI: 7–13 weeks) (*n* = 53 in expansion cohort) mOS: 52 weeks (95% CI: 40–72 weeks) (*n* = 42 in BS), 49 weeks (95% CI: 34–73 weeks) (*n* = 42 in STS), 57 weeks (95% CI: 33–86 weeks) (*n* = 60 in expansion cohort)	15 ≥ G3 TRAE in BS 19 ≥ G3 TRAE in STS cohort 19 ≥ G3 TRAE in expansion cohort
		NCT02691026	Phase II (terminated due to slow enrollment as a result of low incidence of MPNST and the COVID-19 pandemic)	MPNST	NA	NA
		NCT03012620	Phase II (active, not recruiting)	CNS neoplasm, germ cell/embryonal neoplasms, neuroendocrine carcinoma, NK/T cell lymphoma, ovarian neoplasm, sarcoma, thyroid cancer	1 CR, 14 PR, 33 SD (*n* = 98) mPFS: 2.75 months (*n* = 98 in overall), 7.5 months (ASPS), 6.6 months (chordoma), 2.1 months (DSRCT) mOS: 19/7 months (*n* = 98 in overall), 10 months (DSRCT)	NA
		NCT03013127	Phase II (terminated due to poor clinical benefits)	OGS	9 PD with no clinical benefit after 18 weeks of treatment (*n* = 12) mPFS: 1.7 months (95% CI: 1.2–2.2 months) mOS: 6.6 months (95% CI: 3.8–9.3 months)	0 ≥ G3 TRAE
		NCT03316573	Phase II (suspended due to low accrual)	Follicular dendritic cell sarcoma, histiocytic sarcoma, interdigitating dendritic cell sarcoma, lymphoma	NA	NA
		NCT03469804	Phase II (active, not recruiting)	Classic and endemic Kaposi sarcoma	2 CR, 10 PR, 5 SD, 71% BORR (95%CI: 44–90%) (*n* = 17)	2 ≥ G3 TRAE
	Olaparib	NCT05156268	Phase II (recruiting)	Endometrial carcinosarcoma	NA	NA
	Olaratumab	NCT03126591	Phase I (completed)	STS	0 CR, 6 CR, 9 SD (*n* = 28) mPFS: 2.7 months (95% CI:1.3–4.07 months) mOS: 14.8 months (95% CI: 12.6 months – NR)	≥ G3 TRAE in 2 patients reported
	± Pazopanib	NCT05679921	Phase II (not yet recruiting)	STS	NA	NA
	RT	NCT03338959	Phase I/II (active, not recruiting)	STS	NA	NA
	+ RT or SOC alone	NCT03092323	Phase II (recruiting)	STS	NA	NA
	T-VEC	NCT03069378	Phase II (active, not recruiting)	Cutaneous angiosarcoma, epithelioid sarcoma, MFS, UPS (expansion cohort)	43% BORR (95%CI: 0.1–0.82) (*n* = 7 in cutaneous angiosarcoma cohort), 0% BORR (*n* = 3 in epithelioid sarcoma), 11% BORR (95% CI: 0.0–0.48) (*n* = 9 in MFS/UPS cohort) mPFS: 54 weeks (95% CI: 3 weeks – NR in cutaneous angiosarcoma cohort), NA in cutaneous angiosarcoma cohort, 14.9 weeks (95% CI: 7–110 weeks in MFS/UPS cohort)	1 ≥ G3 TRAE in cutaneous angiosarcoma cohort
	Ziv-Aflibercept	NCT02298959	Phase I (active, not recruiting)	Advanced cancer, sarcoma	No DLT reported Melanoma: 1 CR, 1 PR Mesothelioma: 1 PR RCC: 1 PR mOS: 3.3 months (CRC), (90% CI: 0.6–3.4 months), NR (melanoma), 12.5 months (ovarian), (90% CI: 3.8–13.6 months), NR (others), 15.7 months (RCC) (90% CI: 2.5–15.7 months),	G3 TRAE reported in 19 patients (*n* = 33)
± Pembrolizumab	Bevacizumab ± pegcetacoplan	NCT04919629	Phase II (recruiting)	Fallopian tube carcinosarcoma, primary peritoneal cancer, recurrent ovarian, fallopian tube cancer	NA	NA
	BT-001	NCT04725331	Phase I/II (recruiting)	Sarcoma, solid tumors	NA	NA
	Eribulin mesylate	NCT05619913	Phase II (recruiting)	Ovarian carcinosarcoma, uterine carcinosarcoma	NA	NA
	GI-101 ± lenvatinib or RT	NCT04977453	Phase I/II (recruiting)	Advanced solid tumors, sarcoma	1 PR (*n* = 16 in GI-101 monotherapy), 2 PR (*n* = 9 in GI-101 + pembrolizumab arm)	≥ G3 TRAE reported in 3 patients in GI-101 monotherapy arm No ≥ G3 TRAE reported in GI-101 + pembrolizumab arm
	KVA12123	NCT05708950	Phase I/II (recruiting)	Sarcoma, solid tumors	NA	NA
	MQ719	NCT05859074	Phase I (recruiting)	Kaposi sarcoma, solid tumors	NA	NA
	Mupadolimab ± or ciforadenant	NCT03454451	Phase I (active, not recruiting)	Advanced cancer, sarcoma	No objective responses by RECIST criteria were observed (*n* = 34)	28 ≥ G3 TRAE
	Nanatinostat + valganciclovir	NCT05166577	Phase I/II (recruiting)	EBV + LMS, EBV + sarcoma, EBV + solid tumors	NA	NA
	RT	NCT05488366	Phase I (recruiting)	STS	NA	NA
	T3011	NCT04370587	Phase I/II (recruiting)	HNSCC, melanoma, NSCLC, sarcoma, solid tumor, squamous cell carcinoma	No DLT reported	No treatment related serious adverse events reported
	LY3435151	NCT04099277	Phase I (terminated due to strategic business decision)	LMS, solid tumors, UPS	NA	NA
Pembrolizumab/nivolumab	Autologous HER2 CAR T cells	NCT04995003	Phase I (recruiting)	HER2 + sarcoma	NA	NA
Retifanlimab	Docetaxel + gemcitabine	NCT04577014	Phase I/II (recruiting)	STS	17% ORR (95% CI: 1%-64%) and 50% (95%: 19%-81%) in the run in (*n* = 7) and de-escalation (*n* = 6) cohort, 100% DCR (95% CI: 52%-100%)	11 ≥ G3 TRAE
± Retifanlimab	Doxorubicin + ifosfamide	NCT04968106	Phase II (recruiting)	Resectable sarcoma	NA	NA
Sintilimab	Doxorubicin hydrochloride + ifosfamide	NCT04356872	Phase II (unknown)	DDLPS, myxoid liposarcoma, UPS, SS	62.5% ORR (*n* = 24)	1/6 DLT
	Surufatinib + RT	NCT05839275	Phase Ib/II (recruiting)	High risk localized STS	NA	NA
Spartalizumab	NA	NCT04802876	Phase II (active, not recruiting)	PD-1-high mRNA expressing tumors, sarcoma	NA	NA
Toripalimab	NA	NCT03474640	Phase I (active, not recruiting)	Advanced malignancies, chondrosarcoma, STS	NA	NA

*AIDS* Acquired immunodeficiency syndrome, *ASPS* Alveolar soft part sarcoma, *BORR* Best overall response rate, *BS* Bone sarcoma, *CAB-AXL-ADC* Conditionally active biologic AXL-targeted antibody drug conjugate, *CAR* Chimeric antigen receptor, *CI* Confidence interval, *CNS* Central nervous system, *CR* Complete response, *CRC* Colorectal cancer, *CTLA4* Cytotoxic T-lymphocyte-associated protein 4, *DCR* Disease control rate, *DDLPS* Dedifferentiated liposarcoma, *DLT* Dose-limiting toxicity, *DSRCT* Desmoplastic small round cell tumor, *EBV* Epstein-Barr virus, *ES* Ewing sarcoma, *GIST* Gastrointestinal stromal tumor, *HER2* Human epidermal growth factor receptor 2, *HIV* Human immunodeficiency virus, *HNSCC* Head and neck squamous cell carcinoma, *HR* Hazard ratio, *IFN-γ-1β* Interferon-γ-1β, *IgG* Immunoglobulin G, *INI1* Integrase interactor 1, *IQR* Interquartile range, *irAEs* Immune-related adverse events, *LMS* Leiomyosarcoma, *LPS* Liposarcoma, *MFS* Myxofibrosarcoma, *mOS* Median overall survival, *mPFS* Median progression-free survival, *MPNST* Malignant peripheral nerve sheath tumor, *mRNA* Messenger ribonucleic acid, *NA* Not available, *NCT* National Clinical Trial, *NK cells* Natural killer cells, *NR* Not reached, *NSCLC* Non-small cell lung cancer, *NY-ESO-1* New York Esophageal Squamous Cell Carcinoma 1 gene, *OGS* Osteosarcoma, *ORR* Objective response rate, *PD-1* Programmed cell death 1, *PD-L1* Programmed death-ligand 1, *PD* Progressive disease, *PR* Partial response, *RCC* Renal cell carcinoma, *RT* Radiotherapy, *SABR* Stereotactic ablative radiotherapy, *SBRT* Stereotactic body radiation therapy, *SCLC* Small cell lung cancer, *SD* Stable disease, *SOC* Standard of care, *SS* Synovial sarcoma, *STS* Soft-tissue sarcoma, *TIL* Tumor infiltrating lymphocyte, *TNBC* Triple-negative breast cancer, *TRAE* Treatment-related adverse event (G3 = grade 3), *T-VEC* Talimogene Laherparepvec, *UPS* Undifferentiated pleomorphic sarcoma

**Fig. 1:**
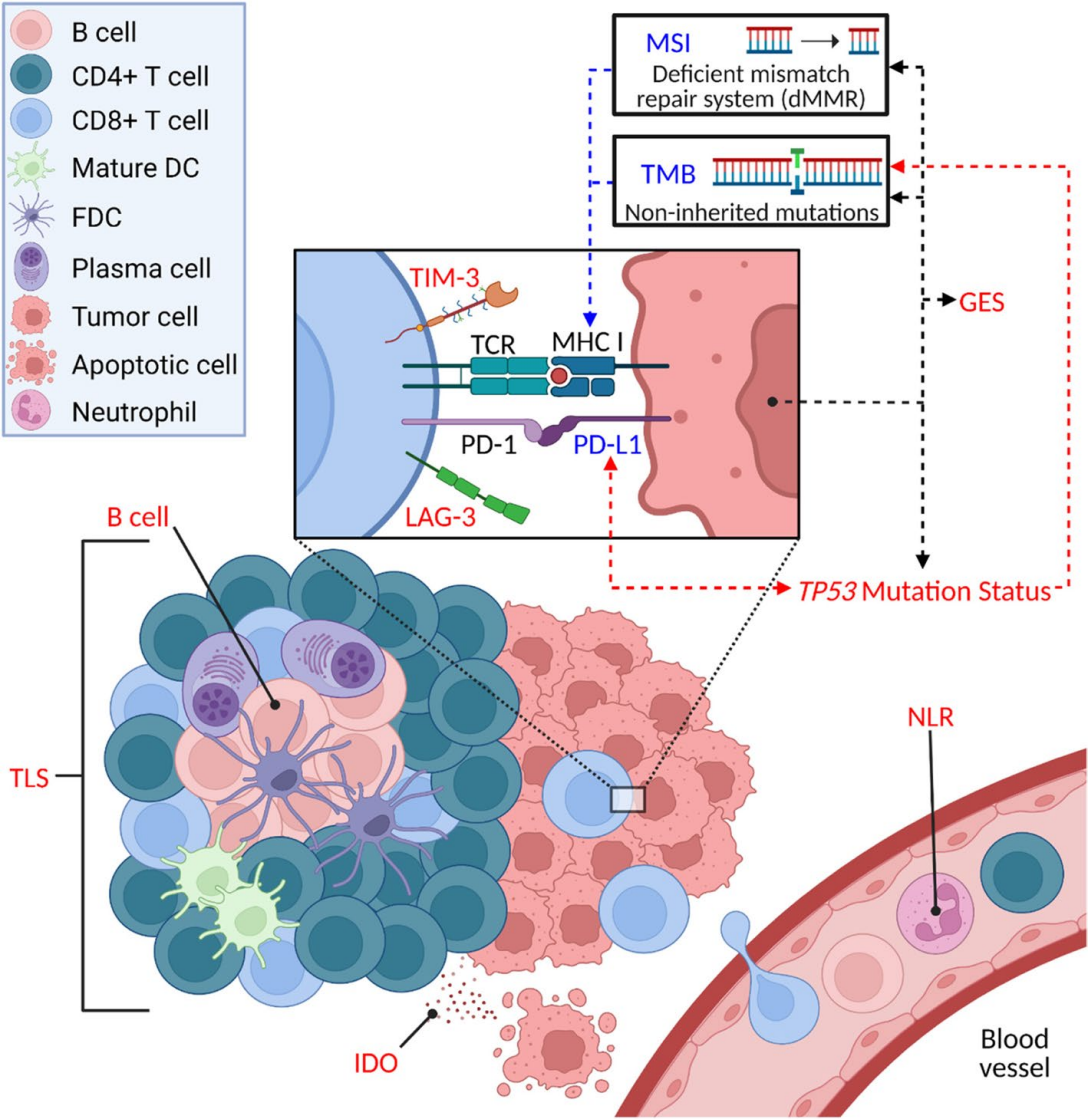
Overview of approved and exploratory biomarkers for immune checkpoint inhibitors (ICIs) in cancer. Tumor and immune features can influence response to ICIs and serve as predictive biomarkers for response. FDA- and EMA-approved biomarkers for ICIs in cancer are indicated in blue, while exploratory biomarkers are indicated in red. MSI and a high TMB contribute to the expression of tumor neoantigens presented by MHC I molecules on tumor cells that can be recognized by the TCR on CD8^+^ T cells, leading to antitumor T cell activity. In gastrointestinal cancers, the expression of immunogenic neoantigens in tumors with high TMB is dependent on certain mutational signatures [41]. On the other hand, binding of PD-L1 on tumor cells to PD-1 on T cells leads to the suppression of T cell antitumor activity. Additionally, exhausted T cells may also express the exhaustion markers TIM-3 and LAG-3. In lung adenocarcinoma, *TP53* mutations are correlated with higher TMB and neoantigen expression, while *TP53* missense but not nonsense mutations are associated with increased PD-L1 expression [42]. Various GES have also been associated with response to ICIs. IDO contributes to T cell suppression and its expression was induced in resistant HCC after ICI therapy [43]. The presence of B cells and TLS have been associated with improved prognosis and response to ICIs in several cancers, including sarcomas. Within the blood, a higher baseline circulating NLR has also been found to correlate with poorer outcomes in patients receiving ICIs in lung cancer [44].

In this review, we will consider existing US FDA-approved and European Medicines Agency (EMA)-approved biomarkers for ICIs in clinical practice and evaluate their applicability in sarcomas. We then discuss exploratory biomarkers and evidence for their potential utility in sarcomas. Predictive biomarkers covered in this review are illustrated in Fig. 1.

## Biomarkers approved for immune checkpoint inhibition in cancer

ICI therapy is indicated without biomarker requirement in several cancer settings because of studies demonstrating improved clinical outcomes [45]. These indications include patients with advanced melanoma [46–48], relapsed or refractory Hodgkin lymphoma [49, 50], cisplatin-ineligible patients with urothelial carcinoma [49, 50], patients with relapsed or refractory primary mediastinal large B-cell lymphoma [51, 52], second-line treatment for patients with HCC [49, 53], patients with Merkel cell carcinoma [49, 53], patients with recurrent or metastatic HNSCC [24, 54] and Bacillus Calmette-Guérin-unresponsive high risk non-muscle invasive bladder cancer [55]. In contrast, there are cancer types such as sarcoma [35], breast, prostate and colon cancers [56] that demonstrate lower frequency of response to ICI therapy, and would therefore require biomarkers to distinguish between responders and non-responders.

Currently, only three predictive biomarkers have been approved by the FDA for ICI therapy in cancers, namely programmed death-ligand 1 (PD-L1), microsatellite instability (MSI) or defective mismatch repair (dMMR), and tumor mutational burden (TMB), while only two predictive biomarkers, namely PD-L1 and MSI/dMMR have been approved by the EMA [57]. Variability in the antibody clones, expression thresholds, scoring systems and the cell types expressing PD-L1 among FDA/EMA-approved PD-L1 assays across multiple cancer types can pose difficulty of interpretation for researchers and clinicians. PD-L1 assays were previously described by Wang et al. to have poor diagnostic accuracy, poor predictability, and low negative predictive value in cancers [58], also limiting its clinical use in sarcomas. For the detection of MSI-high (MSI-H) tumors, approved assay methods include immunohistochemistry (IHC), polymerase chain reaction (PCR) and whole exome sequencing (WES). Both IHC and PCR are established methods and are widely available in the pathology laboratory. However, IHC is limited by its low analytic sensitivity and accuracy, while PCR may be unable to capture full MSI profiles that results in missing 0.3% to 10% of MSI-H cases [58, 59]. Circumventing the limitations of PCR, WES provides better predictive power compared to PCR and can be used for all tumor types [58]. Additionally, TMB can be derived from WES and may provide a better prediction of response to ICIs [58]. On the other hand, WES is characterized by high cost, limited availability, potentially complicated pipelines and requires technical expertise that may hinder its clinical utility [60]. Table 2 summarizes FDA- and EMA-approved predictive biomarkers for ICIs in selected cancers.

**Table 2:** Overview of Food and Drug Administration (FDA)- and European Medicines Agency (EMA)-approved predictive biomarkers for patient selection for immune checkpoint inhibition

**Predictive Biomarkers**	**Assay Methods**	**Antibody**	**Expression Threshold**	**Cancers**	**Regulatory Authority**	**NCT Number**	**Author, Year**
PD-L1	PD-L1 IHC 22C3 pharmDx assay	Monoclonal mouse anti PD-L1 clone 22C3	PD-L1 CPS ≥ 20 and CPS ≥ 1	TNBC	FDA	NCT02622074	Schmid et al., 2020 [61]
			CPS ≥ 1	HNSCC	FDA/EMA	NCT02358031	Burtness et al., 2019 [62]
			TPS ≥ 50%	NSCLC	FDA/EMA	NCT02142738	Reck et al., 2019 [63]
			CPS ≥ 10	UC	EMA	NCT02256436	Bellmunt et al., 2017 [64]
	PD-L1 IHC 28–8 pharmDx assay	Monoclonal rabbit anti PD-L1 clone 28–8	TPS ≥ 1%	NSCLC	FDA/EMA	NCT02477826	Hellmann et al., 2019 [65]
	VENTANA SP142 PD-L1 IHC assay	Monoclonal rabbit anti PD-L1 clone SP142	IC ≥ 1%	TNBC	FDA/EMA	NCT02425891	Schmid et al., 2018 [30]
			TC ≥ 50% or IC ≥ 10%	NSCLC	FDA	NCT02008227	Rittmeyer et al., 2017 [66]
			IC ≥ 5%	UC	FDA/EMA	NCT02108652	Rosenberg et al., 2016 [67]
	VENTANA SP263 assay	Monoclonal rabbit anti PD-L1 clone SP263	TC ≥ 25% or IC ≥ 25%	UBC	FDA	NCT01693562	Massard et al., 2016 [65, 68]
MSI	PCR or IHC	-	MSI-H/dMMR	Colorectal cancer	FDA/EMA	NCT02460198	Le et al., 2020 [69]
	Fluorescent Multiplex PCR-based method	-	MMR-deficient or proficient	Progressive metastatic carcinomas	FDA	NCT01876511	Le et al., 2015 [70]
TMB	FoundationOne CDx assay	-	tTMB-high ≥ 10 mutations per Mb	Advanced solid tumors	FDA	NCT02628067	Marabelle et al., 2020 [71]
	WES	-	NA	Advanced solid tumors	FDA	NCT02054806	Ott et al., 2019 [72]

Year = year of publication

*CPS* Combined positive score, *dMMR* Deficient mismatch repair, *HNSCC* Head and neck squamous cell carcinoma, *IC* Percentage of tumor-infiltrating immune cells within the tumor area expressing PD-L1, *IHC* Immunohistochemistry, *MMR* Mismatch repair, *MSI* Microsatellite instability, *MSI-H* Microsatellite instability-high, *NCT* National Clinical Trial, *NSCLC* Non-small cell lung cancer, *PCR* Polymerase chain reaction, *PD-1* Programmed cell death 1, *PD-L1* Programmed death-ligand 1, *TC* Percentage of tumor cells within total tumor cells expressing PD-L1, *TMB* Tumor mutational burden, *TNBC* Triple-negative breast cancer, *TPS* Tumor proportion score, *tTMB* Tissue tumor mutational burden, *UBC* Urothelial bladder cancer, *UC* Urothelial carcinoma, *WES* Whole exome sequencing

### Programmed death-ligand 1 (PD-L1)

PD-L1 is a ligand for the T cell immune checkpoint receptor programmed cell death 1 (PD-1) and is expressed by a variety of normal and immune cells. Interaction between PD-1 and PD-L1 serves to promote self-tolerance through the suppression of T cell activation. Cancer cells have been found to exploit the PD-1/PD-L1 axis for immune evasion through the overexpression of PD-L1 [73]. Thus, PD-1 and PD-L1 expression provide an attractive avenue to predict response to ICI therapy. At present, there are four FDA- and three EMA-approved PD-L1 assays (Table 2). For further reading, a detailed review on the key parameters for the FDA-approved PD-L1 assays has been conducted by Wang et al*.,* describing different test methods and challenges [58].

The diverse and dynamic PD-L1 expression on specific cell types within the tumor microenvironment (TME) has made the correlation of global PD-L1 expression with response to ICI therapy challenging. Noguchi et al. demonstrated that PD-L1 expression in tumor-associated macrophages are partially dependent on interferon-γ (IFN-γ) [74]. Further studies by Lau et al. in PD-L1-depleted mouse models highlighted that although immune evasion occurs at a repressed rate, infiltrating myeloid cells may contribute to immune evasion through compensatory PD-L1 expression [75]. There is also contradicting evidence demonstrating that efficacy of PD-L1 blockade is independent of PD-1/PD-L1 expression on tumor cells [76]. Instead, PD-L1 expression on dendritic cells (DCs) and macrophages correlates to clinical response in melanoma and ovarian cancer patients [76]. Given that PD-L1 expression level in the TME is highly variable, global PD-L1 positivity alone may not be sufficient to predict response to ICIs [77]. Instead, understanding the effects of differential expression of PD-L1 in specific immune and tumor cells in the TME may reveal mechanisms of the PD-1/PD-L1 axis that could be exploited to better predict response to ICI therapy.

**Fig. 2:**
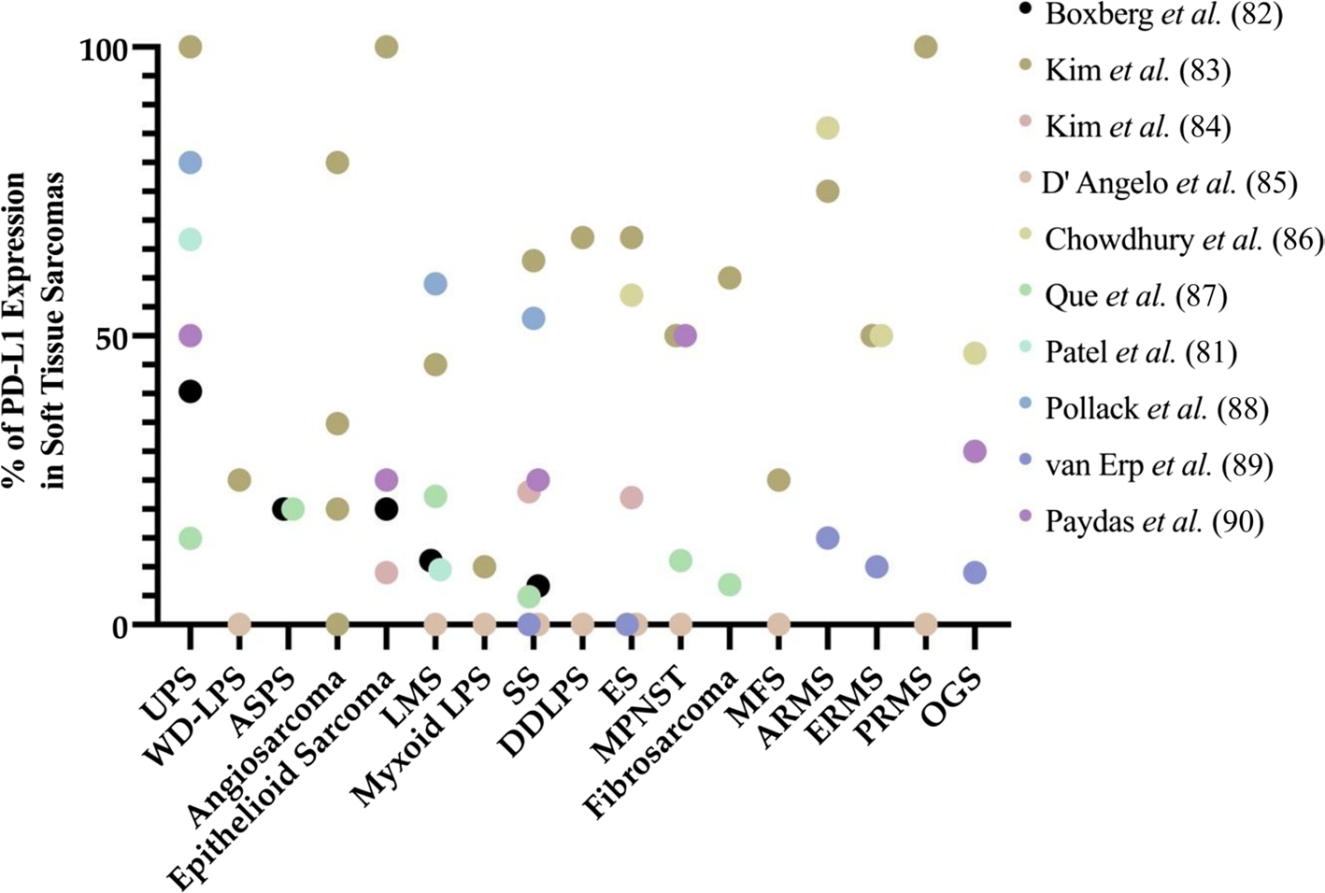
Prevalence of PD-L1 expression in soft-tissue sarcomas across published studies. This figure shows the levels of PD-L1 expression in different sarcoma subtypes that has been reported across a number of studies [79, 81–90]. Inter- and intra-variability of PD-L1 expression among different sarcoma subtypes warrants extensive studies to establish the use of existing PD-L1 assays as a reliable predictive biomarker to immune checkpoint inhibition in soft tissue sarcomas (STS). ARMS: Alveolar rhabdomyosarcoma; ASPS: Alveolar soft part sarcoma; DDLPS: Dedifferentiated liposarcoma; ERMS: Embryonal rhabdomyosarcoma; ES: Ewing sarcoma; LMS: Leiomyosarcoma; LPS: Liposarcoma; MFS: Myxofibrosarcoma; MPNST: Malignant peripheral nerve sheath tumor; OGS: Osteosarcoma; PRMS: Pleomorphic rhabdomyosarcoma; SS: Synovial sarcoma; UPS: Undifferentiated pleomorphic sarcoma; WD-LPS: Well differentiated liposarcoma

In sarcomas, PD-L1 expression levels have shown conflicting association with ICI response [78]. Indeed, levels of PD-L1 expression can vary widely between different histological subtypes [79] (Fig. 2) that is further complicated by the heterogenous TME present in primary and metastatic lesions [78, 80]. This high degree of heterogeneity in PD-L1 expression, coupled with limited studies clarifying the relationship between PD-L1 expression and response to ICI warrants further investigation of the use of PD-L1 testing in sarcomas. Additionally, Patel et al. demonstrated that pre-treatment with radiotherapy (RT) prior to surgical resection increased PD-L1 expression in 10.9% of patient STS tumors (*p* = 0.056) while post-operative radiation therapy did not elicit PD-L1 expression in any STS resection samples [81]. These findings suggest that PD-L1 expression can be influenced by other treatment modalities, though much work remains to be done due to the small study sample sizes and limited studies available in sarcomas.

### Microsatellite Instability (MSI)/ Deficient Mismatch Repair (dMMR)

MSI occurs when dMMR results in hypermutation in short stretches of DNA (microsatellites). MSI-H have higher potential to code for tumor-associated neoantigens [91] that can be recognized by the immune system, eliciting an antitumor response. A phase II study by Le et al. demonstrated that high levels of somatic mutations in dMMR colorectal tumors was associated with increased expression of tumor-associated antigens compared to proficient mismatch repair (pMMR) colorectal tumors [70]. In the same study, 40% of patients with dMMR tumors responded to PD-1 inhibition, while none of the patients with pMMR tumors achieved an objective response, thus highlighting the role of dMMR as a predictive biomarker for ICI response.

Currently, IHC, PCR and next-generation sequencing (NGS) are used to assess MSI [92]. In the same review mentioned previously, Wang et al*.* has provided a comprehensive evaluation of the three assays in use [58].

**Fig. 3:**
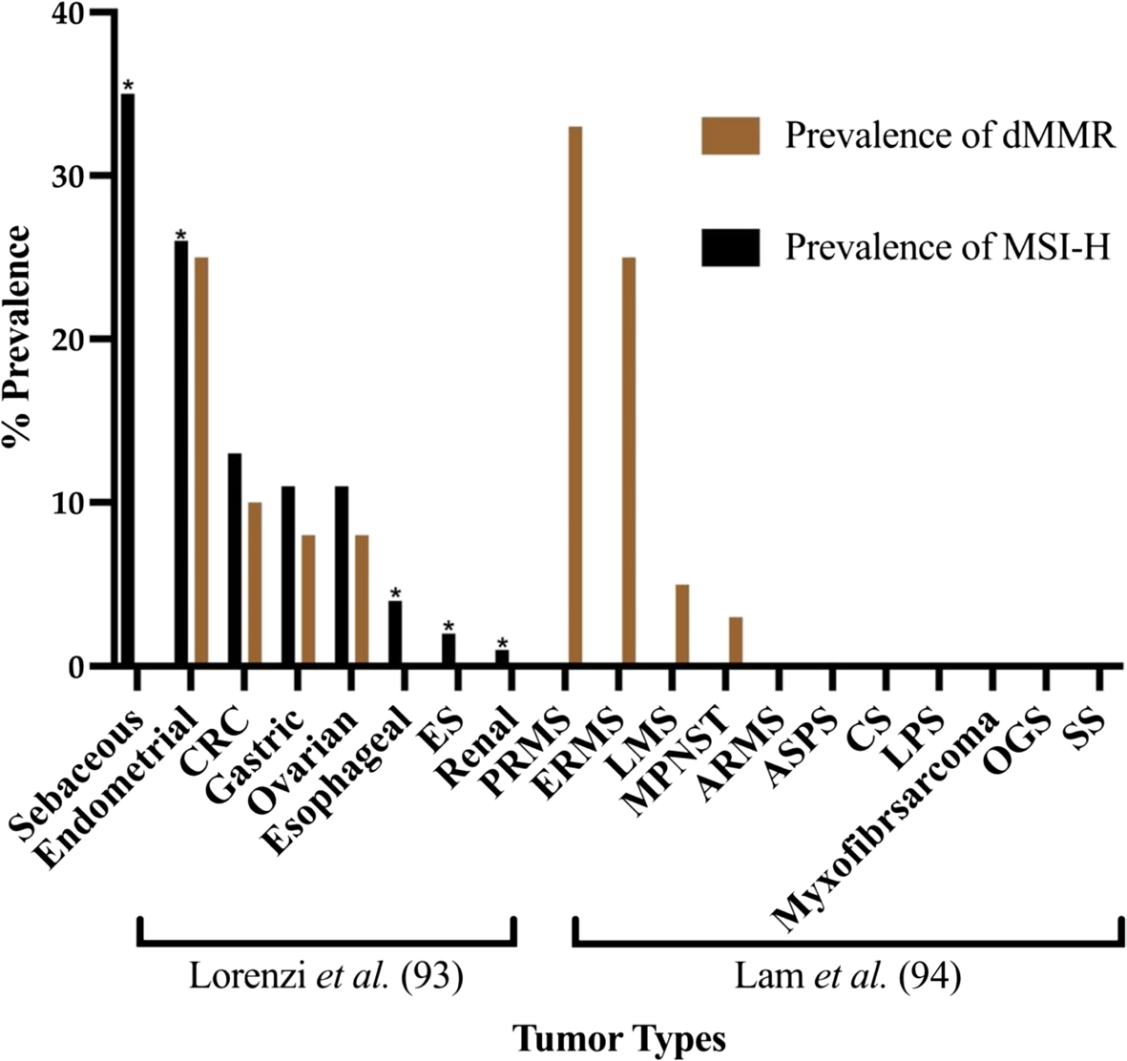
Pooled prevalence of MSI-H and dMMR among different tumor types. Bar graphs show the prevalence of MSI-H and dMMR in various cancers as summarized by Lorenzi et al. and Lam et al. [93, 94]. Low prevalence of MSI-H in Ewing sarcoma (ES) and wide variation of dMMR between sarcoma subtypes warrants further studies to explore the correlation between MSI-H / dMMR and clinical response to immune checkpoint inhibition. Results from Lorenzi et al*.* were pooled from various studies. Lam et al*.* did not evaluate for MSI-H. Asterisk indicates analysis for dMMR was not feasible. ARMS: Alveolar rhabdomyosarcoma; ASPS: Alveolar soft part sarcoma; CRC: Colorectal cancer; CS: Chondrosarcoma; ERMS: Embryonal rhabdomyosarcoma; ES: Ewing sarcoma; LMS: Leiomyosarcoma; MPNST: Malignant peripheral nerve sheath tumor; OGS: Osteosarcoma; PRMS: Pleomorphic rhabdomyosarcoma; SS: Synovial sarcoma. Asterisk indicates analysis for dMMR was not included

A meta-analysis by Lorenzi et al. reported the prevalence of dMMR among six common tumor types, including colorectal, endometrial, esophageal, gastric, renal and ovarian cancers, which suggested that the prevalence of dMMR/MSI differs between tumor types and cancer stages [93] (Fig. 3). Notably, MSI/dMMR accounts for only approximately 1% of sarcomas, with the exception of pleomorphic rhabdomyosarcoma (PRMS), embryonal rhabdomyosarcomas (ERMS), LMS and malignant peripheral nerve sheath tumor (MPNST) that have higher rates of MSI/dMMR [94]. Given the low prevalence of MSI-H tumors in sarcomas and the lack of trials evaluating the role of MSI in predicting ICI treatment response in sarcomas, MSI/dMMR may be of limited use in guiding the clinical decision-making for ICIs in sarcomas.

### Tumor Mutation Burden (TMB)

Cancer neoantigens are tumor-specific antigens that arise from genetic mutations within tumor cells that can be recognized by the immune system. Hence, highly mutated tumors are more likely to express neoantigens and provide an opportunity for ICIs to reinvigorate the immune system and stimulate an antitumor response [95]. As predicted, improved survival after ICI treatment was indeed observed in patients with high TMB in multiple cancer types [96, 97].

However, the use of high TMB as a predictive biomarker for ICI response has demonstrated conflicting results in gastrointestinal cancers, with most studies reporting the lack of a significant association between high TMB and response to ICIs [71, 98–101]. A retrospective study by Wang et al. analyzed the mutational signatures of microsatellite-stable gastrointestinal tumors with high TMB and found that not all genes associated with high TMB correlated with an enhanced antitumor response, hence suggesting that the types of mutational signatures in tumors could play a role in the expression of immunogenic neoantigens [41].

TMB is defined as the number of somatic mutations in the tumor exome [96] and can be classified into low (1–5 mutations per Mb), intermediate (6–19 mutations per Mb) and high (≥ 20 mutations per Mb) [102]. TMB can be measured using WES, but clinical implementation has been limited due to the large amount of genomic deoxyribonucleic acid (DNA) required, long sequencing time, availability of matched samples and costs [103]. To circumvent the limitations of WES, targeted NGS panels have been developed to accurately recapitulate WES-derived genomic information while sequencing less DNA [60, 96, 104]. In assessing TMB, both WES and targeted NGS panels can be influenced by various factors from sample collection, processing, sequencing, data analysis to the lack of harmonization in reporting cut-offs, thus limiting the independent clinical utility of TMB [58].

Studies analyzing genomic profiles in sarcomas have suggested low somatic mutation burden across most sarcomas. A study of the molecular landscape of adult STS demonstrated an average of 1.06 mutations per Mb across 206 sarcomas of different histological subtypes [105], while genomic profiling of over 6,100 sarcoma cases showed a median of 1.7 mutations per Mb [106]. Additionally, even in dMMR sarcomas, TMB appears lower than that in other dMMR tumor types, with a median TMB of 16 mutations per Mb compared to 28 mutations per Mb [107]. The exception appears to be head and neck angiosarcomas, where 63.4% of cases have high TMB defined as ≥ 10 mutations per Mb [108]. Even so, in a phase II clinical trial of metastatic or unresectable angiosarcoma treated with combined ipilimumab and nivolumab (NCT02834013), the objective response rate (ORR) was only 25% and six-month progression-free survival (PFS) was 38% [109].

Overall, the lack of studies examining the use of TMB as a predictive biomarker of ICI response in sarcomas, poor stratification of TMB classification, as well as a low median TMB across most sarcomas may limit the clinical utility of TMB in directing ICI use in sarcomas.

## Exploratory biomarkers for immune checkpoint inhibition in sarcomas

In this section, we discuss eight exploratory biomarkers that may predict response to ICI therapy in sarcomas, including gene expression signatures (GES), circulating neutrophil-to-lymphocyte ratio (NLR), indoleamine 2,3-dioxygenase (IDO), lymphocyte activation gene 3 (LAG-3), T cell immunoglobulin and mucin domain-containing protein 3 (TIM-3), *TP53* mutation status, B cells, and tertiary lymphoid structures (TLS).

### Gene Expression Signatures (GES)

GES are presented as a group of genes whose differential expression has been found to be associated with a particular outcome, and have been used in the determination of diagnosis, prognosis, and the prediction of therapeutic outcomes [110]. Methods used to measure gene expression levels include ribonucleic acid (RNA) microarray and RNA sequencing [111, 112], as well as newer methods including single-cell RNA sequencing, single-nucleus RNA sequencing [113] and spatial transcriptomics [114].

In several cancers, various GES have been found to be capable of predicting ICI response, including in melanoma [115–117], NSCLC [118–121], gastric cancer [122], lower-grade glioma [123] and some across multiple cancer types such as in both NSCLC and melanoma [124]. In addition, a pan-tumor signature predictive of ICI response was derived from 220 patients across HNSCC, gastric cancer, triple-negative breast cancer, bladder, anal canal, biliary, colorectal, esophageal, and ovarian cancers. This pan-tumor signature defined by Ayers et al. contains IFN-γ- and T cell-associated inflammatory genes, and high expression of this gene signature correlated well with objective response to pembrolizumab (1-sided *p*-value < 0.001) [125].

In STS, given the heterogeneity in genomic alterations across the various histological subtypes [126], identifying a robust GES that is able to be used in multiple subtypes may prove to be challenging. Nonetheless, Petitprez et al. identified a B lineage signature associated with improved response to ICI therapy in STS [127], and this will be discussed in further detail in the section on B cells below.

Presently, the implementation of routine gene sequencing is costly, and the complexity of its results require expertise to analyze and interpret before they can be used to guide clinical decision making [128, 129]. There is a thus a need to identify a GES with minimal number of genes to be sequenced in order to determine response to ICIs, with its accuracy subsequently being validated in a prospective trial.

### Circulating Neutrophil-to-Lymphocyte Ratio (NLR)

Compared to other biomarkers that may require patients’ tumor samples, NLR can be easily derived from whole blood as a less invasive procedure with minimal risk of complications. The ease of sample acquisition and minimal patient risk has led to extensive studies of its use in cardiovascular diseases, infectious diseases, and cancers where it has been found to correlate with prognosis [130].

In the published literature, there is a lack of clearly defined cutoffs as well as contrasting evidence for the use of NLR across and within the different cancer types [131]. In a retrospective study of 509 patients with advanced cancer, a non-linear response trend during ICI treatment was observed and significant decreases or increases in NLR on-treatment correlated to poorer prognostic outcomes [132]. Conversely, in a meta-analysis by Jing et al., higher NLR at baseline across 23 studies correlated to lower OS in lung cancer patients receiving ICIs [44]. In STS, Strong et al. found that high baseline NLR, defined as ≥ 4.5, was not independently associated with worse survival outcomes in patients with extremity STS [133]. On the other hand, Chan et al. used receiver operating curve analysis to determine a cutoff of high NLR at > 2.5, and demonstrated high baseline NLR to be an independent marker for poor prognosis in STS patients [134].

Overall, while the use of NLR in the clinic is less invasive and more convenient, the lack of harmonization in key parameters such as a standardized baseline NLR may hinder the use of NLR as a predictor of response to ICIs in sarcomas. The establishment of clearly defined cutoffs would be essential to support its use.

### Indoleamine 2,3-Dioxygenase (IDO)

IDO is a heme-containing enzyme that catalyzes the conversion of tryptophan into kynurenine. IDO contributes to an immunosuppressive effect involving both CD4^+^ and CD8^+^ T cells via the rapid depletion of tryptophan [135]. Subsequent downstream activation of stress response mediator general control nonderepressible 2 (GCN2) kinase results in cell cycle arrest [136], thus inhibiting T cell proliferation. Additionally, IDO has been demonstrated to upregulate regulatory T cell (T_reg_) activation and activity [137, 138]. Thus, IDO has been suggested for use as a prognostic marker.

In a meta-analysis by Wang et al., high expression of IDO in tumor tissues was associated with poor prognosis (pooled hazard ratio (HR) 1.92, 95% CI, 1.52–2.43, *p* < 0.001) and tumor progression (pooled HR = 2.25, 95% CI, 1.58–3.22, *p* < 0.001) in cancer patients [135]. An in vitro study has also shown that ICI therapy induces IDO in resistant HCC through upregulation of IFN-γ that consequently results in adaptive immune evasion [43]. These studies shed light on alternative immune evasion pathways conferred in the TME.

In sarcomas, Hiroshi et al. analyzed 47 patient specimens in which 96% of high-grade osteosarcoma of the extremities are IDO-positive [139]. Consequently, IDO positivity has been correlated to decreased progression free survival (PFS) (*p* = 0.016) and OS (*p* = 0.005) [139]. To circumvent IDO-induced resistance, IDO inhibitors have been proposed to be included in combination treatment with ICIs. Imatinib, a tyrosine kinase inhibitor used in the treatment of gastrointestinal stromal tumor (GIST), has demonstrated inhibition of IDO expression in GIST mouse models [140]. However, clinical trials testing for combination treatment with ipilimumab and imatinib demonstrated limited efficacy and antitumor immune response in GISTs [141].

In conclusion, IDO has been recognized as an immune target in the TME, and the combination of IDO inhibitors with ICIs has also shown efficacy in several phase I/II clinical trials [142]. However, the phase III trial of epacadostat with pembrolizumab in unresectable or metastatic melanoma (NCT02752074) failed to demonstrate better efficacy versus placebo and pembrolizumab [143]. Taken together, there is a need for deeper understanding of the role that IDO plays in the TME before establishing IDO as a biomarker.

### Lymphocyte-activation gene 3 (LAG-3)

In March 2022, the FDA approved a LAG-3 ICI (relatlimab) given in combination with the PD-1 inhibitor nivolumab, expanding the list of immunotherapeutic options in advanced melanoma [144]. LAG-3 is an inhibitory molecule expressed by activated T cells and associates with the T cell receptor (TCR) and CD3 at the T cell surface [145]. The intracellular region of LAG-3 is responsible for transducing inhibitory signals to suppress T cell activation, but the molecular mechanisms governing this remain under investigation [146]. The known ligands of LAG-3 include major histocompatibility complex (MHC) class II [147, 148], galectin-3 [149] and fibrinogen-like protein 1 (FGL1) [150]. The utility of LAG-3 ICIs remains to be seen, but an early phase I/II study of combination treatment with LAG-3 and PD-1 inhibitor showed synergistic activity albeit with modest antitumor response [151]. For further reading, Huo et al. recently reviewed the clinical development of these novel agents [152], which will not be further elaborated on in this review.

In STS, analysis of blood samples from patients and healthy donors found that LAG-3 expression in peripheral T cells was correlated with the degree of intratumoral CD8^+^ T cell infiltration and poor prognosis [153]. Due to the novelty of anti-LAG-3 antibodies, there have been limited clinical trials regarding the use of LAG-3 as a potential immune biomarker for ICI response. As ongoing and future research uncovers more about the role of LAG-3 in suppressing T cell activation and the molecular mechanisms governing this, we would then be able to better understand its place in cancer immunotherapy and as a predictive biomarker for ICI response in sarcomas.

### T-cell immunoglobulin and mucin domain-containing protein 3 (TIM-3)

TIM-3 is an immune checkpoint receptor that has been found to be expressed on many types of immune cells, including CD4^+^ and CD8^+^ T cells [154], T_reg_ cells [155], myeloid cells [156], natural killer (NK) cells [157] and mast cells [158, 159]. In CD8^+^ T cells, co-expression of TIM-3 and PD-1 has been observed on the most exhausted subset of tumor-infiltrating lymphocytes [160, 161].

TIM-3 has several ligands that bind to different regions on the receptor, including galectin-9 (Gal-9), phosphatidylserine, high mobility group protein B1 (HMGB1) and carcinoembryonic antigen-related cell adhesion molecule 1 (CEACAM1) [159]. Gal-9 is expressed and secreted by many hematopoietic cells and some tumor cells, and its binding has been reported to result in T cell inhibition and cell death [159, 162]. HMGB1 binds to DNA from dying cells and is also secreted by tumor cells. HMGB1 binding to DNA facilitates their uptake and activation of toll-like receptors (TLRs), but it can also be bound by TIM-3, which sequesters it and prevents its activation of TLRs, thereby dampening antitumor immunity [159, 163]. CEACAM1 is expressed by T cells [164], DCs [165], monocytes [166] and macrophages [167], and its binding results in TCR signaling inhibition [164].

In mouse models of lung adenocarcinoma, Koyama et al. observed that in tumors which progressed following initial response to anti-PD-1 therapy, there was an upregulation of other immune checkpoint receptors, particularly TIM-3, on PD-1 antibody-bound T cells. Subsequent administration of combined anti-PD-1 and anti-TIM-3 therapy resulted in improved survival. The upregulation of TIM-3 was also seen in two patients who developed adaptive resistance to anti-PD-1 therapy, presenting TIM-3 upregulation as a possible biomarker of PD-1 therapy resistance [168].

Several anti-TIM-3 antibodies are being tested in phase I/II clinical trials, with some in combination with anti-PD-1/-PD-L1 antibodies, in the contexts of acute myelogenous leukemia, myelodysplastic syndrome, and various solid tumors. This combination has been demonstrated to be generally well-tolerated in early data and some anti-TIM-3 antibodies have displayed activity in lung cancer [169]. Nonetheless, the efficacy of these novel agents remains to be explored in sarcomas.

There have also been some studies evaluating the prognostic value of TIM-3 expression. Zang et al*.* demonstrated that TIM-3 was an independent prognostic indicator for poor OS in patients with malignant tumors (HR = 1.54; 95% CI, 1.19–1.98; *p* = 0.001) based on multivariate Cox regression analysis of 28 studies, and this was also observed in The Cancer Genome Atlas (TCGA) patient cohorts (HR = 1.2; *p* < 0.001). When stratified by tumor type, however, TIM-3 expression was not associated with OS in sarcoma (3 studies with 780 cases; *p* = 0.232) [170]. In contrast, Pu et al*.* reported that among 38 osteosarcoma tumor samples, 36 samples expressed TIM-3, and TIM-3 overexpression was associated with poorer OS (*p* < 0.001) [171].

Overall, anti-TIM-3 targeted therapy is still in its early stages of development, and more robust data on TIM-3 is needed to evaluate its role as a predictive biomarker for ICI therapy in sarcomas. Clinical trials evaluating the efficacy of anti-PD-1/PD-L1 antibodies combined with anti-TIM-3 antibodies could uncover more information on the relationship between immune checkpoint receptors within the TME.

### *TP53* mutation status

The tumor suppressor protein p53 is critical in the prevention of oncogenesis [172]. *TP53* is the most frequently mutated gene among human cancers [172–174] and *TP53* mutations commonly result in both loss of tumor suppressor function and gain of oncogenic function [175].

In sarcomas, *TP53* is also one of the most frequently altered genes, albeit widely varying across histological subtypes [42, 127, 176–178]. Nassif et al*.* reported that *TP53* mutation in sarcomas is associated with shorter disease-free survival (HR = 1.63; 95% CI, 1.04–2.54; Cox *p* = 0.032) and better treatment outcomes with anthracyclines (OR = 3.70; 95% CI, 1.20–11.97; *p* = 0.02) [42, 176, 177, 179, 180]. However, there has been a lack of studies evaluating the use of *TP53* as an immune biomarker for ICI therapy in sarcomas.

Nevertheless, *TP53* mutation status has been observed to be significantly correlated with PD-L1 expression [42] and response to ICI therapy in NSCLC [181–184]. In NSCLC and colorectal cancer (CRC), Agersborg et al*.* explored the relationship between mutation profile and PD-L1 expression and found that tumors with *TP53* mutation in the NSCLC cohort had significantly higher PD-L1 expression (*p* = 0.01), though this was not observed in the CRC cohort (*p* = 0.5). In fact, the CRC cohort had significantly lower expression of PD-L1 (*p* = 0.0005) compared to the NSCLC cohort despite similar rates of *TP53* mutation across both cancers, suggesting that varying mechanisms regulate PD-L1 expression across different tumor types [185].

In addition, Sun et al*.* compared lung adenocarcinoma TMB data of *TP53*-missense-mutant and *TP53*-nonsense-mutant groups to *TP53*-wild-type groups from Memorial Sloan Kettering Cancer Center (MSKCC) (*p* < 0.01 and *p* < 0.05 respectively), TCGA (*p* < 0.0001 for both) and GENE + (*p* < 0.0001 for both) databases using a Wilcoxon test and reported that both *TP53*-mutant groups demonstrated elevated TMB and neoantigen levels compared to the *TP53*-wild-type group [42].

Taken together, *TP53* mutation status appears to be correlated with other biomarkers of ICI therapy in NSCLC. However, whether this is also true in sarcoma remains to be seen, as further investigation into the relationship between *TP53* mutation status and response to ICIs is needed.

### B Cells

B cells are responsible for the humoral arm of the adaptive immune system. Activation of naïve B cells by CD4^+^ T cells results in B cell proliferation, somatic hypermutation of immunoglobulin genes and class switching. Subsequently, activated B cells differentiate into plasmablasts and long-lived plasma cells which produce antigen-specific antibodies that are responsible for the clearance of antigens [186].

The role of B cells in the TME remains controversial, with conflicting evidence across different studies. A comprehensive review of publications investigating the prognostic value of tumor-infiltrating B cells in cancer found that 50% of studies reported a positive prognostic effect for B cells, while 9% and 40% reported a negative or neutral effect respectively [187]. An in vitro study showed that B cells suppress tumor immunity by downregulating the expression of IFN-γ in CD8^+^ T cells, a cytokine possessing antitumor activity [188], while increasing interleukin-10 (IL-10) production that further inhibits IFN-γ production by T cells [189]. Interestingly, co-culture of B cells with different cancer cell lines yielded different expression levels of IL-10, with sarcoma cells failing to stimulate IL-10 production in B cells, in contrast to Friend murine leukemia virus gag-expressing and melanoma cells which induced B cell IL-10 secretion [189]. In contrast, a separate study highlighted the antibody-mediated antitumor response of activated B cells in murine models of metastatic pulmonary tumors [190]. These conflicting reports of the role of B cells in antitumor immunity are likely due to heterogeneity of the B cell population within the TME, which could ultimately influence clinical outcomes.

Various subtypes of B cells are found in the TME. In tertiary lymphoid structures (TLS) within the TME, B cells are thought to be mainly involved in antigen presentation, where they help to activate both CD4^+^ and CD8^+^ T cells [191–194]. Subsequent antigen-driven maturation of B cells into plasma cells leads to the generation of in situ tumor antigen-specific antibodies [191]. Thus, B cells are instrumental in the generation of antitumor activity initiated within TLS. An immunosuppressive subset of B cells within the TME has also been described, commonly referred to as regulatory B cells. These cells act by secreting immunosuppressive cytokines [189] and have been identified in the TME of several cancers, including breast cancer [195], HCC [196], tongue squamous carcinoma [197], gastric cancer [198] and prostate cancer [199].

Increasing numbers of studies on immune subsets in the TME have led to the development of predictive biomarkers focused on the B cell compartment. In melanoma and RCC, B cell markers were enriched in tumors from responders versus non-responders to ICI therapy [178]. In another study involving the gene expression analysis of 3585 patients, a B cell-related gene signature comprising nine cytokine signaling genes was predictive of clinical response to ICI therapy in melanoma [200].

In STS, Petitprez et al*.* identified the overexpression of the B lineage signature as a distinctive feature of an immune class of sarcomas with high immune infiltration (*p* = 1.8 × 10^–29^) and found that it was also significantly associated with improved OS (*p* = 4.25 × 10^–4^). Patients in this immune class also demonstrated the best response to pembrolizumab defined by the percentage change in size of target lesions from baseline (*n* = 45, *p* = 0.026) in the SARC028 trial [127].

In conclusion, the role that B cells play in the TME is not clearly understood, given the numerous B cell subtypes present. Nonetheless, there is evidence for B cells playing a crucial role in response to ICI therapy in sarcomas and other cancers, as seen from the B cell-related gene signatures. Characterization of B cell subtypes in the TME as well as further validation of these gene signatures in larger cohorts and prospective trials could help identify the specific B cell populations and their cell states as a predictor for response to ICIs.

### Tertiary Lymphoid Structures (TLS)

TLS are ectopic lymphoid structures that have been found to develop in response to chronic inflammation [201] and in various solid tumor types [202, 203]. Within the cancer literature, definitions of what constitutes a TLS as well as its maturation state vary significantly. Sautès-Fridman et al*.* and Vanhersecke et al*.* defined TLS as lymphoid aggregates consisting of B lymphocytes that are closely associated with plasma cells and T lymphocytes, making the distinction that mature TLS (mTLS) have at least one CD23^+^ follicular dendritic cell, while immature TLS (iTLS) are CD23^−^ [201, 204]. In contrast, Lin et al*.* classified TLS into two categories based on their morphology – TLS aggregates, which are simply small clusters of lymphocytes; and TLS follicles, which are large clusters of lymphocytes that can be further distinguished based on the presence or absence of germinal centers [205].

TLS have been found to benefit prognosis [204–207] and are also associated with favorable ICI treatment outcomes [127, 204, 208–211] in several cancers. In a retrospective analysis of patient samples comprising 11 different tumor types from three independent cohorts by Vanhersecke et al., a higher proportion of patients with mTLS demonstrated objective response to ICIs compared to patients with iTLS or no TLS (36.9% versus 19.3% versus 19%, respectively, *p* = 0.015). Importantly, mTLS were predictive of response to ICIs regardless of PD-L1 expression [204]. Remarkably, in the phase II PEMBROSARC trial (NCT02406781) cohort, TLS-positive patients (*n* = 30) demonstrated a 6-month non-progression rate (NPR) and ORR of 40% (95% CI, 22.7–59.4) and 30% (95% CI, 14.7–49.4) respectively, compared to a 6-month NPR and ORR of 4.9% (95% CI, 0.6–16.5) and 2.4% (95% CI, 0.1–12.9) respectively, in the unselected all-comer cohorts [210]. Interestingly, in the study by Petitprez et al*.* mentioned in the previous section, at least one TLS was found in the TME of nine out of eleven tumors (82%) in the immune-high class of STS [127]. Taken together, this class of tumors is characterized by a high expression of the B lineage signature and the presence of TLS, further supporting the significance of the role that B cells and TLS play in the TME.

This significant improvement in clinical benefit highlights the potential for the presence of TLS to be utilized as a biomarker for the selection of patients with STS for ICI therapy.

Although TLS are emerging as key players in the TME, the exact mechanisms of their antitumor activity have not been fully elucidated. It has been proposed that TLS provide a favorable environment for antigen presentation and the differentiation and proliferation of lymphocytes in the TME as well as the generation of effector memory T cells, memory B cells and plasma cells [191, 201, 205]. In some TLS, spatial visualization through IHC has shown that B cells in TLS express markers of germinal center B cells, including activation-induced deaminase, the proliferation marker Ki67 and transcription factor B-cell lymphoma 6 (BCL6) [212]. The expression of these markers suggests an ongoing humoral immune response generated within TLS.

The growing evidence for TLS predicting response to ICI therapy thus gives rise to the important question of whether their use as predictive biomarkers can be implemented in clinical workflows. This will be discussed in the following section.

## Clinical relevance of TLS as a predictive biomarker for ICI response in sarcomas

Of all the exploratory predictive biomarkers for response to ICI in sarcomas, the presence of TLS appears most promising thus far based on the results from the PEMBROSARC trial [210] and the study by Petitprez et al*.* [127]. However, the identification of TLS via multiplex IHC involves a complex laboratory workflow that requires substantial runtime and is not available in most pathology laboratories. As such, several automated methodologies have been suggested to simplify the workflow for TLS identification.

Panagiotis et al*.* described the use of a deep learning algorithm to quantitatively identify hematoxylin and eosin (H&E)-stained TLS [213]. The proposed computational methodology has accurately identified TLS comparable to a human counterpart and circumvents TLS that may not be identified by specific IHC staining in lung cancer [213]. However, the algorithm is not without limitations, as it does not discriminate between the various maturation states of TLS described in the literature [204, 213]. Nevertheless, preliminary identification of TLS through digital pathology provides a novel option to incorporate into the clinical workflow.

Subsequently, downstream processes to characterize TLS can include various immunostaining techniques such as multiplex IHC and immunohistofluorescence (IHF) [214]. Currently, there is a lack of standardized marker panels to robustly quantify TLS [201]. Vanhersecke et al*.* adopted a previously described method consisting of H&E, CD3 and CD20 staining to assess the preliminary TLS status of pathological samples [127], followed by a 5-marker multiplex IHF panel consisting of CD4, CD8, CD20, CD21 and CD23 to differentiate between CD23-positive mTLS and CD23-negative iTLS [204]. Similarly, the phase II PEMBROSARC trial cohort screened for TLS using H&E, CD3 and CD20 staining [127], followed by three different multiplex IHF panels to visualize the immune environment of TLS [210]. Other studies have suggested the use of genomic probes to identify the presence of TLS in melanoma through a 12-chemokine gene signature [215].

Although screening with a wide coverage of immune markers could improve sensitivity and specificity in TLS detection, using more markers for every patient sample would also inevitably translate to increased costs and turnaround time which would not be ideal in the clinical setting. Additionally, the lack of standardized immune markers in TLS detection could lead to inconsistencies in the identification of TLS in the clinic. Hence, there is an urgent need to streamline and define a standardized panel of markers that can be adopted in the clinical setting.

It is important to also take into consideration that the presence of TLS alone may not always be able to predict response to ICIs due to the complex interplay of factors within the TME. For example, tumors may have innate resistance to ICIs, or even acquire resistance after treatment. Jenkins et al*.* attributed ICI treatment failure to three broad causes – inadequate formation of antitumor T cells, impaired function of tumor-specific T cells, or impaired formation of memory T cells [216]. Hence, the use of biomarkers to infer the states of immune cells in the TME together with the presence or absence of TLS may be able to better predict response to ICIs.

## Conclusion

Presently in sarcomas, there is still a lack of robust predictive biomarkers that can be implemented in the clinic. Putative biomarkers will need to be tested in clinical trials to establish their roles in the treatment of sarcomas using ICIs. As new mechanisms emerge, this list will also expand, but it is also critically important that tests are simple and cost-effective with a short turnaround time, so as to be applicable in centers worldwide. Patients matched to biomarkers that accurately predict response to ICI will change the paradigm for systemic treatment in sarcomas and likely supersede the current standard of care.

## Data Availability

Not applicable.

## Abbreviations

AIDS: Acquired immunodeficiency syndrome
ARMS: Alveolar rhabdomyosarcoma
ASPS: Alveolar soft-part sarcoma
BCL6: B-cell lymphoma 6
BORR: Best overall response rate
BS: Bone sarcoma
CAB-AXL-ADC: Conditionally active biologic AXL-targeted antibody drug conjugate
CAR: Chimeric antigen receptor
CEACAM1: Carcinoembryonic antigen-related cell adhesion molecule 1
CI: Confidence interval
CNS: Central nervous system
CPS: Combined positive score
CR: Complete response
CRC: Colorectal cancer
CS: Chondrosarcoma
CTLA4: Cytotoxic T-lymphocyte-associated protein 4
DCR: Disease control rate
DCs: Dendritic cells
DDLPS: Dedifferentiated liposarcoma
DLT: Dose-limiting toxicity
dMMR: Defective mismatch repair
DNA: Deoxyribonucleic acid
DSRCT: Desmoplastic small round cell tumor
EBV: Epstein-Barr virus
EMA: European Medicines Agency
ERMS: Embryonal rhabdomyosarcoma
ES: Ewing sarcoma
FDA: Food and Drug Administration
FGL1: Fibrinogen-like protein 1
Gal-9: Galectin-9
GCN2: General control nonderepressible 2
GES: Gene expression signatures
GIST: Gastrointestinal stromal tumor
H&E: Hematoxylin and eosin
HCC: Hepatocellular carcinoma
HER2: Human epidermal growth factor receptor 2
HIV: Human immunodeficiency virus
HMGB1: High mobility group protein B1
HNSCC: Head and neck squamous cell carcinoma
HR: Hazard ratio
IC: Percentage of tumor-infiltrating immune cells within the tumor area expressing PD-L1
ICI: Immune checkpoint inhibitor
IDO: Indoleamine 2,3-dioxygenase
IFN-γ: Interferon-γ
IFN-γ-1β: Interferon-γ-1β
IgG: Immunoglobulin G
IHC: Immunohistochemistry
IHF: Immunohistofluorescence
IL-10: Interleukin-10
INI1: Integrase interactor 1
IQR: Interquartile range
irAEs: Immune-related adverse events
iTLS: Immature tertiary lymphoid structures
LAG-3: Lymphocyte activation gene 3
LMS: Leiomyosarcoma
LPS: Liposarcoma
MHC: Major histocompatibility complex
MFS: Myxofibrosarcoma
mOS: Median overall survival
mPFS: Median progression free survival
MPNST: Malignant peripheral nerve sheath tumor
mRNA: Messenger ribonucleic acid
MSI: Microsatellite instability
MSI-H: Microsatellite instability-high
MSKCC: Memorial Sloan Kettering Cancer Center
mTLS: Mature tertiary lymphoid structures
NA: Not available
NCT: National Clinical Trial
NGS: Next generation sequencing
NK cells: Natural killer cells
NLR: Neutrophil-to-lymphocyte ratio
NPR: Non-progression rate
NR: Not reached
NSCLC: Non-small cell lung cancer
NY-ESO-1: New York Esophageal Squamous Cell Carcinoma 1 gene
OGS: Osteosarcoma
ORR: Objective response rate
OS: Overall survival
PCR: Polymerase chain reaction
PD-1: Programmed cell death 1
PD-L1: Programmed death ligand 1
PD: Progressive disease
PFS: Progression free survival
pMMR: Proficient mismatch repair
PR: Partial response
PRMS: Pleomorphic rhabdomyosarcoma
RCC: Renal cell carcinoma
RNA: Ribonucleic acid
RT: Radiotherapy
SABR: Stereotactic ablative radiotherapy
SBRT: Stereotactic body radiation therapy
SCLC: Small cell lung cancer
SD: Stable disease
SOC: Standard of care
SS: Synovial sarcoma
STS: Soft-tissue sarcoma
TC: Percentage of tumor cells within total tumor cells expressing PD-L1
TCGA: The Cancer Genome Atlas
TCR: T cell receptor
TIL: Tumor-infiltrating lymphocyte
TIM-3: T cell immunoglobulin and mucin domain-containing protein 3
TLR: Toll-like receptor
TLS: Tertiary lymphoid structures
TMB: Tumor mutational burden
TME: Tumor microenvironment
TNBC: Triple-negative breast cancer
TPS: Tumor proportion score
TRAE: Treatment-related adverse event
T_reg_ cells: Regulatory T cells
tTMB: Tissue tumor mutational burden
T-VEC: Talimogene Laherparepvec
UBC: Urothelial bladder cancer
UC: Urothelial carcinoma
UPS: Undifferentiated pleomorphic sarcoma
US: United States
WD-LPS: Well-differentiated liposarcoma
WES: Whole exome sequencing
